# Evaluation of extravascular lung water and cardiac function in normal vaginal delivery by intrapartum bedside ultrasound

**DOI:** 10.1186/s12884-023-06201-4

**Published:** 2024-01-02

**Authors:** Shi-jie Zhang, Shao-zheng He, Jing-jing Wu, Yong-jian Chen, Guo-rong Lyu

**Affiliations:** 1 Department of Ultrasound, Second Affiliated Hospital of Fujian Medical University, No. 34 North Zhongshan Road, Quanzhou, 362000 Fujian Province China; 2 Department of Obstetrics and Gynecology, Second Affiliated Hospital of Fujian Medical University, Quanzhou, Fujian Province China; 3 Department of Clinical Medicine, Quanzhou Medical College, Quanzhou, Fujian Province China

**Keywords:** Echocardiography, Lung ultrasound, Labor, Cardiac function, Pulmonary edema

## Abstract

**Background:**

Healthy parturients may experience pulmonary edema and disturbed cardiac function during labor. We aimed to evaluate the extravascular lung water (EVLW), intravascular volume, and cardiac function of normal parturients during spontaneous vaginal delivery by bedside ultrasound. And to explore the correlation between EVLW and intravascular volume, cardiac function.

**Methods:**

This was a prospective observational study including 30 singleton-term pregnant women undergoing spontaneous vaginal delivery. Bedside ultrasound was performed at the early labor, the end of the second stage of labor, 2 and 24 h postpartum, and 120 scanning results were recorded. EVLW was evaluated by the echo comet score (ECS) obtained by the 28-rib interspaces technique. Inferior vena cava collapsibility index (IVC-CI), left ventricle ejection fraction, right ventricle fractional area change, left and right ventricular E/A ratio, and left and right ventricular index of myocardial performance (LIMP and RIMP) were measured. Measurements among different time points were compared, and the correlations between ECS and other measurements were analyzed.

**Results:**

During the spontaneous vaginal delivery of healthy pregnant women, 2 had a mild EVLW increase at the early labor, 8 at the end of the second stage of labor, 13 at 2 h postpartum, and 4 at 24 h postpartum (*P* < 0.001). From the early labor to 24 h postpartum, ECS first increased and then decreased, reaching its peak at 2 h postpartum (*P* < 0.001). IVC-CI first decreased and then increased, reaching its minimum at the end of the second stage of labor (*P* < 0.001). RIMP exceeded the cut-off value of 0.43 at the end of the second stage of labor. ECS was weakly correlated with IVC-CI (*r*=-0.373, *P* < 0.001), LIMP (*r* = 0.298, *P* = 0.022) and RIMP (*r* = 0.211, *P* = 0.021).

**Conclusions:**

During spontaneous vaginal delivery, the most vital period of perinatal care is between the end of the second stage of labor and 2 h postpartum, because the risk of pulmonary edema is higher and the right ventricle function may decline. IVC-CI can be used to evaluate maternal intravascular volume. The increase in EVLW may be related to the increase in intravascular volume and the decrease in ventricular function.

**Supplementary Information:**

The online version contains supplementary material available at 10.1186/s12884-023-06201-4.

## Background

Pulmonary edema is a life-threatening disease during delivery, and cardiovascular physiologic changes during labor increase the likelihood of pulmonary edema [1–3]. Pulmonary edema in pregnant women will cause significant morbidity and mortality, and is also a common reason for intensive care admission [1, 4]. Early diagnosis of pulmonary edema and accurate evaluation of maternal hemodynamics can prevent maternal morbidity and mortality and intensive care admission [1].

In the incubation period of pulmonary edema, the extravascular lung water (EVLW) accumulates [5]. Lung ultrasound evaluation of EVLW by B-lines can detect increased EVLW earlier than clinical symptoms, abnormal blood oxygen saturation, or oxygenation index [5]. Lung ultrasound can semi-quantitatively evaluate EVLW with good repeatability and consistency [6–8]. Transabdominal ultrasound measuring inferior vena cava (IVC) and generating inferior vena cava collapsibility index (IVC-CI) can assess intravascular volume quickly and non-invasively [9]. Recent studies have shown that the IVC evaluation could also be performed by the transhepatic view when the standard sagittal IVC visualization from the subcostal region is not available [10, 11]. Transthoracic echocardiography can evaluate cardiac function quickly and non-invasively [12]. The above examination can also guide fluid therapy when necessary [13, 14].

Although there seems to be no difference in lung ultrasound patterns between healthy term pregnant women and healthy nonpregnant women, physiological changes during labor in the cardiovascular system may change the balance between vascular fluid hydrostatic pressure, serum colloid osmotic pressure, and capillary permeability, and then increase the incidence of pulmonary edema [8, 15]. Moreover, uterine contractions during labor can increase the blood volume of the systemic circulation, and pain, anxiety, Valsalva maneuvers, and prolonged lying position can increase cardiopulmonary stress [16, 17]. After delivery, the termination of uteroplacental circulation, the relief of IVC, the well-contracted uterus, and the absorption of extravascular fluid will increase the venous return [17, 18]. In addition, studies have shown that an increase in EVLW is associated with an increase in left ventricular end-diastolic pressure, indicating that left ventricular diastolic function can affect EVLW [7]. Therefore, it can be hypothesized that normal healthy parturients may experience an increase in EVLW during and after childbirth, and there may be a correlation between EVLW and intravascular volume, abnormal cardiac function.

However, there are currently few studies exploring intra- and postpartum lung ultrasound in healthy pregnant women [8, 19]. Moreover, there is a lack of research on the changes in EVLW, intravascular volume, and cardiac function of healthy pregnant women undergoing spontaneous vaginal delivery from the early labor to postpartum. This study aimed to use bedside ultrasound to observe the changes in EVLW, IVC-CI, and cardiac function in healthy pregnant women during and after delivery, and to explore the correlation between EVLW and IVC-CI, cardiac function.

## Methods

### Study population

**Fig. 1:**
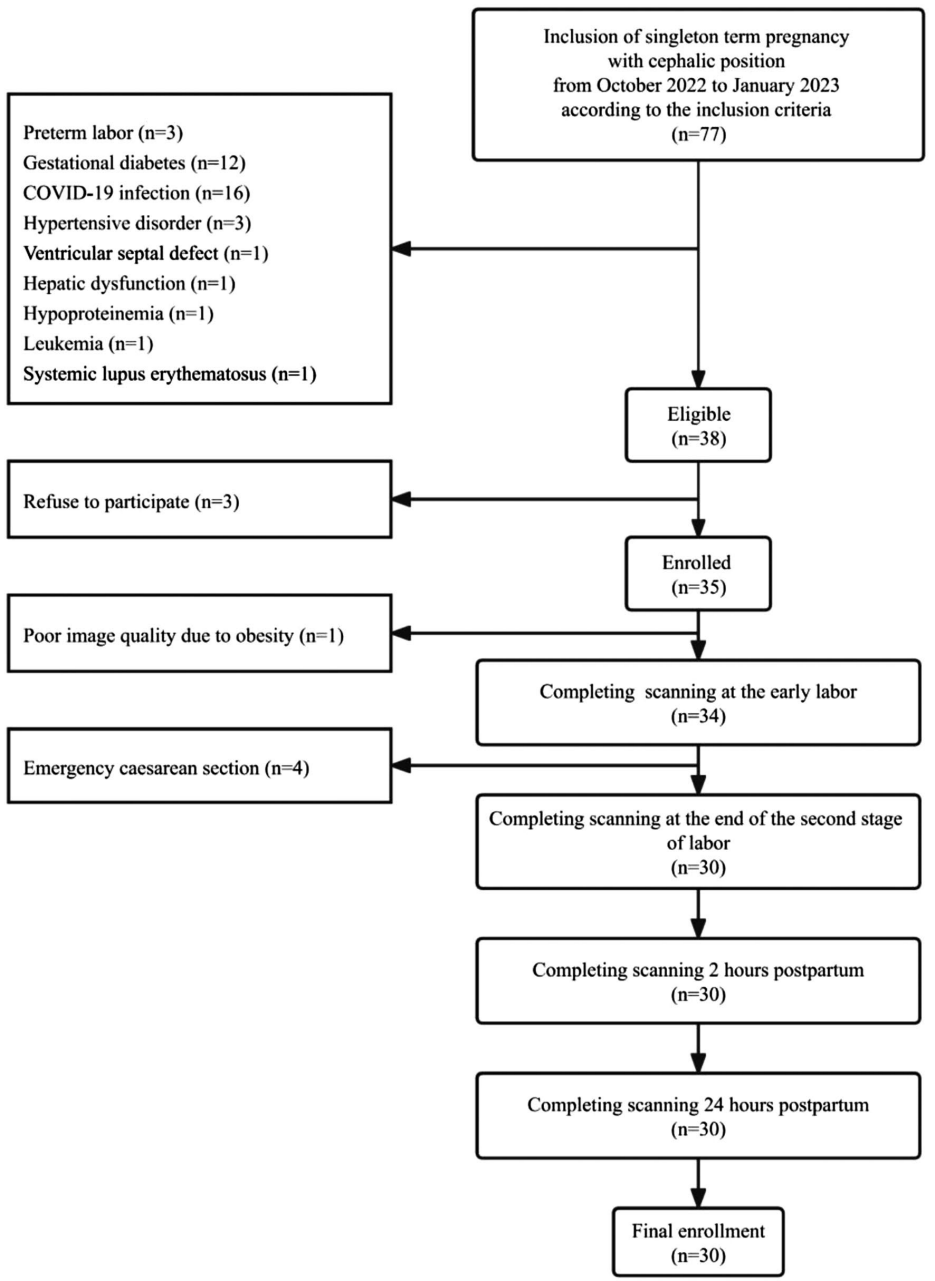
Flow chart of recruitment

This was a prospective observational study performed at a single tertiary perinatal center. A daytime convenience sample of 35 pregnant women from October 2022 to January 2023 was recruited according to the inclusion and exclusion criteria. On arriving at the hospital, each pregnant woman was examined by an obstetrician or a midwife to assess her cervical dilatation and labor stage. Women at the active stage of labor were not considered for inclusion. The electronic medical records of pregnant women were used for the assessment of inclusion and exclusion criteria. The inclusion criteria were singleton term pregnancy with cephalic presentation, spontaneous vaginal delivery, age ≥ 18 years, and ability to understand and provide informed consent. The exclusion criteria were systemic diseases that may lead to pulmonary edema (such as hypertension, heart disease, asthma, pulmonary diseases, immune diseases, and diabetes, etc.), previous or current COVID-19 infection, emergency cesarean section, poor image quality (such as due to obesity, thickened breasts, etc.), postpartum complications that affect respiration or circulation (such as postpartum hemorrhage), and addiction to tobacco, alcohol, or drugs. Finally, 30 pregnant women completed the ultrasound evaluation and 120 records were obtained (Fig. 1). The demographic and obstetric data of the enrolled were shown in Table 1. The perinatal management was performed according to the guidelines [20, 21]. This report follows the guidelines of Strengthening the Reporting of Observational Studies in Epidemiology (STROBE).

**Table 1:** Demographic and obstetric data of 30 enrolled women

Variables	Value
Age, y	29.50 (6.75)
BMI, kg/m^2^	24.86 (1.61)
Nulliparous, n (%)	19(63.33)
Gestational age, wk	39 + 6 (1 + 2)
Total duration of labor, h:min	9:35 (4:46)
First stage of labor, h:min	7:35 (4:48)
Second stage of labor, min	36 (43)
Third stage of labor, min	10 (03)
Oxytocin, n (%)	22 (73.33)
Neuraxial analgesia, n (%)	19 (63.33)
Estimated net fluid intake^*^, ml	-190 (672.50)

Values reported as median (interquartile range) or frequency (%) as appropriate

BMI: body mass index

^*^Net fluid intake = estimated total intravenous fluid intake − (estimated blood loss × 3)

### Ultrasound procedure

All ultrasound examinations were performed by a single researcher (S.Z.) trained in lung ultrasound and echocardiography. A Mindray Z6 bedside scanner (Mindray Bio-Medical Electronics Co., Ltd., Shenzhen) with a 3C5P abdominal transducer (3.5 MHz ~ 6.5 MHz) and a 2P2P cardiac transducer (2 MHz ~ 5 MHz) was used. To observe the longitudinal changes, four time points were selected for continuous recording: the early labor, the end of the second stage of labor, 2 h postpartum, and 24 h postpartum. The early labor was defined as a fully effaced cervix with 3 ~ 4 cm dilation and at least three contractions within 10 min recorded on tocography [22]. All ultrasound records were saved for offline analysis. The lung ultrasound B-lines were independently reviewed by two researchers (S.Z. and Y.C.). In cases of dispute, another researcher (G.L.) was consulted. All researchers had at least 2 years of experience in lung ultrasound diagnosis.

EVLW was evaluated by the echo comet score (ECS) obtained by the 28-rib interspaces technique [6]. The 28-rib interspaces technique involves scanning the midaxillary line, anterior axillary line, midclavicular line, and parasternal line of the left 2 ~ 4th and right 2 ~ 5th intercostal spaces. The total number of B-lines among the 28 intercostal spaces generated the ECS. The B-lines are defined as a high echo line that starts from the pleural line, extends in a laser-like manner to the edge of the screen without attenuation, and slides with breathing [5]. EVLW was classified according to ECS: ECS ≤ 5 are none EVLW, 6 to 15 are mild, 16 to 30 are moderate, and ≥ 31 or diffuse distribution throughout the whole lungs are severe [5].

The long axis of IVC was identified in a subxiphoid view with the woman in a supine position by the cardiac transducer. The inner border diameter of IVC was measured perpendicular to its long axis at the end of expiration (IVCe) and inspiration (IVCi) respectively, just proximal to the junction of the hepatic veins [9]. The IVC-CI was calculated according to the formula: *IVC*-*CI*=[(*IVCe*-*IVCi*)/*IVCe*]×100%.

The echocardiography was performed according to the joint recommendations of the American Society of Echocardiography and the European Association of Cardiovascular Imaging [31]. The left ventricle ejection fraction (LVEF), right ventricle fractional area change (RVFAC), left and right ventricular E/A ratio (LV E/A and RV E/A), and left and right ventricular index of myocardial performance (LIMP and RIMP) were measured. The heart rate was measured before each examination.

### Statistical analysis

Continuous variables were expressed as median and interquartile range. Categorical variables were summarized as frequencies and percentages. Friedman test was used for comparisons of measurements among the early labor, the end of the second stage of labor, 2 h postpartum, and 24 h postpartum. Afterward, a q-test was used for pairwise comparison, and Holm correction was performed. Spearman correlation analysis was used to evaluate the correlation between ECS and other measurements. The correlation coefficient of 0.8 ~ 1.0 was extremely strong, 0.6 ~ 0.8 was strong, 0.4 ~ 0.6 was moderate, 0.2 ~ 0.4 was weak, and 0.0 ~ 0.2 was extremely weak or uncorrelated. Statistical analysis was done using R software (ver. 4.1.3; R Development Core Team, Austria, Vienna), and results with a *P* ≤ 0.05 were considered significant. Since this was a prospective exploratory study and no relevant data were available, no attempt was made to calculate the sample size.

### Ethics statement

The study was approved by the Medical Research Ethics Committee of the Second Affiliated Hospital of Fujian Medical University on August 30, 2022 (No. 2,022,278), and all women included in the study provided written informed consent.

## Results

**Fig. 2:**
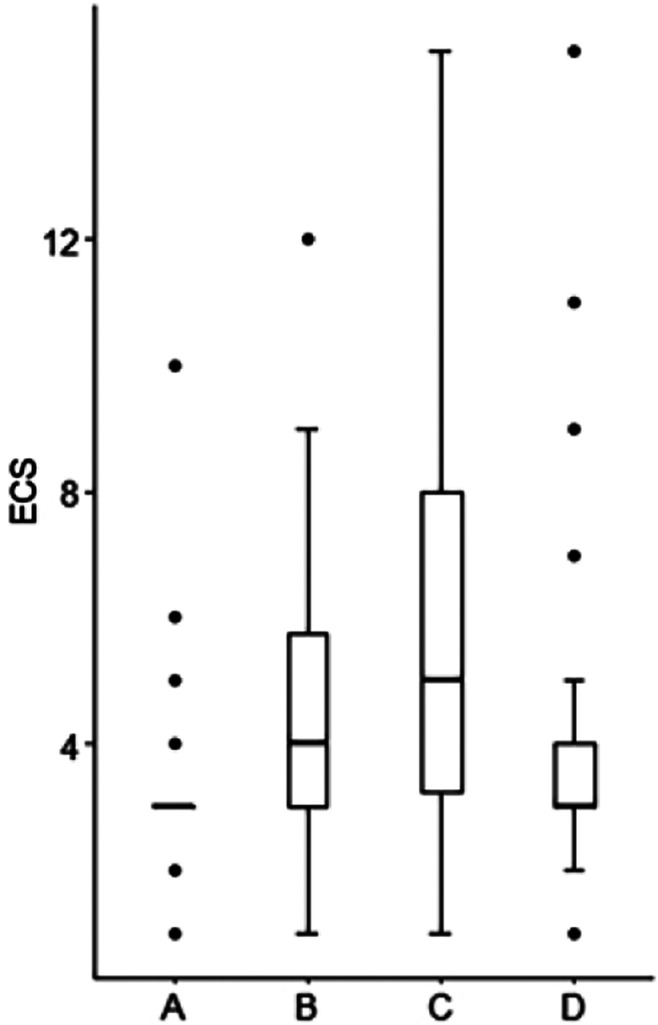
Box plot of ECS during and after labor. ECS: echo comet score. In the abscissa, A: the early labor, B: the end of the second stage of labor, C: 2 h postpartum, D: 24 h postpartum respectively

During the spontaneous vaginal delivery of healthy pregnant women, 2 had a mild EVLW increase at the early labor, 8 at the end of the second stage of labor, 13 at 2 h postpartum, and 4 at 24 h postpartum (*P* < 0.001). From the early labor to 24 h postpartum, ECS first increased and then decreased, reaching its peak at 2 h postpartum (*P* < 0.001) (Fig. 2; Table 2). Figure S1 demonstrated the changes in B lines at four time points for a woman who underwent spontaneous vaginal delivery.

**Table 2:** Examination of lung ultrasound during and after labor

	Early labor	End of the second stage of labor	2 hours postpartum	24 hours postpartum	*P* value
EVLW					
None	28(93.33)	22(73.33)^a^	17(56.67)^a^	26(86.67)^c^	< 0.001
Mild	2(6.67)	8(26.67)^a^	13(43.33)^a^	4(13.33)^c^	< 0.001
Moderate	0(0)	0(0)	0(0)	0(0)	-
Severe	0(0)	0(0)	0(0)	0(0)	-
ECS	3.00(0)	4.00(2.75)^a^	5.00(4.75)^ab^	3.00(1.00)^bc^	< 0.001

Values reported as median (interquartile range) or frequency (%) as appropriate

EVLW: extravascular lung water, ECS: echo comet score

^a^ Compared with the early labor, the difference is statistically significant, and after q test with Holm correction, *P* < 0.05

^b^ Compared with the end of the second stage of labor, the difference is statistically significant, and after q test with Holm correction, *P* < 0.05

^c^ Compared with 2 h postpartum, the difference is statistically significant, and after q test with Holm correction, *P* < 0.05

**Fig. 3:**
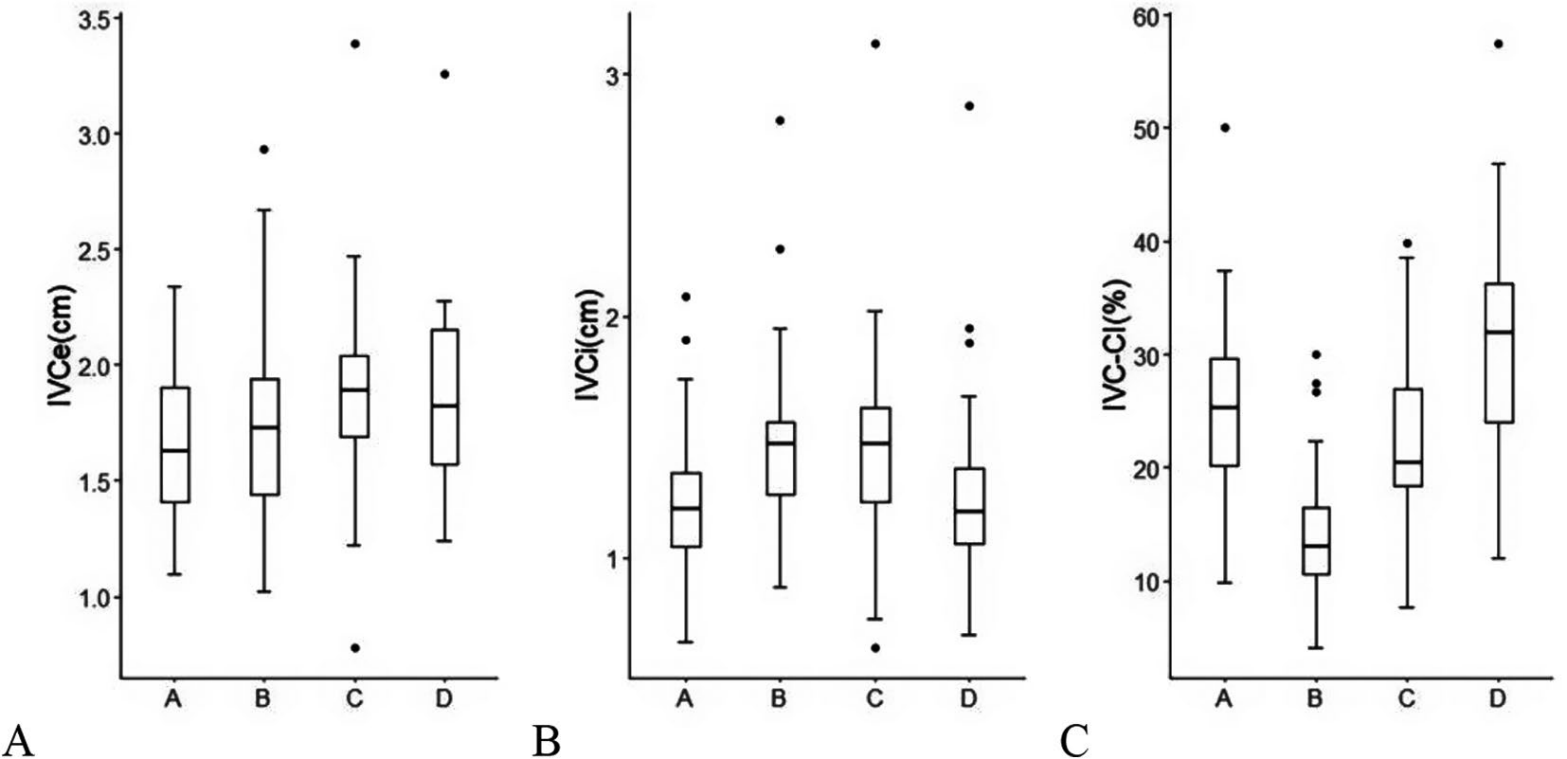
Box plot of IVEe (**A**), IVCi (**B**), and IVC-CI (**C**) during and after labor. IVCe: the diameter of the inferior vena cava at the end of expiration, IVCi: the diameter of the inferior vena cava at the end of inspiration, IVC-CI: inferior vena cava collapsibility index. In the abscissa, A: the early labor, B: the end of the second stage of labor, C: 2 h postpartum, D: 24 h postpartum respectively

IVCe increased at 2 h postpartum (*T* = 2.671, *P* = 0.045) and 24 h postpartum (*T* = 2.779, *P* = 0.040) compared to the early labor (Fig. 3A). From the early labor to 24 h postpartum, IVCi first increased and then decreased, reaching its peak at the end of the second stage of labor and 2 h postpartum (*P* = 0.004) (Fig. 3B). IVC-CI first decreased and then increased, reaching its minimum at the end of the second stage of labor (*P* < 0.001) (Fig. 3C; Table 3).

**Table 3:** Intravascular volume during and after labor

Variables	Early labor	End of the second stage of labor	2 h postpartum	24 h postpartum	*P* value
IVCe, cm	1.63(0.49)	1.73(0.50)	1.89(0.35)^a^	1.82(0.58)^a^	0.023
IVCi, cm	1.21(0.31)	1.47(0.30)^a^	1.47(0.39)^a^	1.19(0.31)^bc^	0.004
IVC-CI, %	25.29(9.44)	12.96(5.88)^a^	20.39(8.59)^b^	31.88(12.26)^abc^	< 0.001

Values reported as median (interquartile range) as appropriate

IVCe: end expiratory inferior vena cava diameter, IVCi: end inspiratory inferior vena cava diameter, IVC-CI: inferior vena cava collapsibility index

^a^ Compared with the early labor, the difference is statistically significant, and after q test with Holm correction, *P* < 0.05

^b^ Compared with the end of the second stage of labor, the difference is statistically significant, and after q test with Holm correction, *P* < 0.05

^c^ Compared with 2 h postpartum, the difference is statistically significant, and after q test with Holm correction, *P* < 0.05

**Fig. 4:**
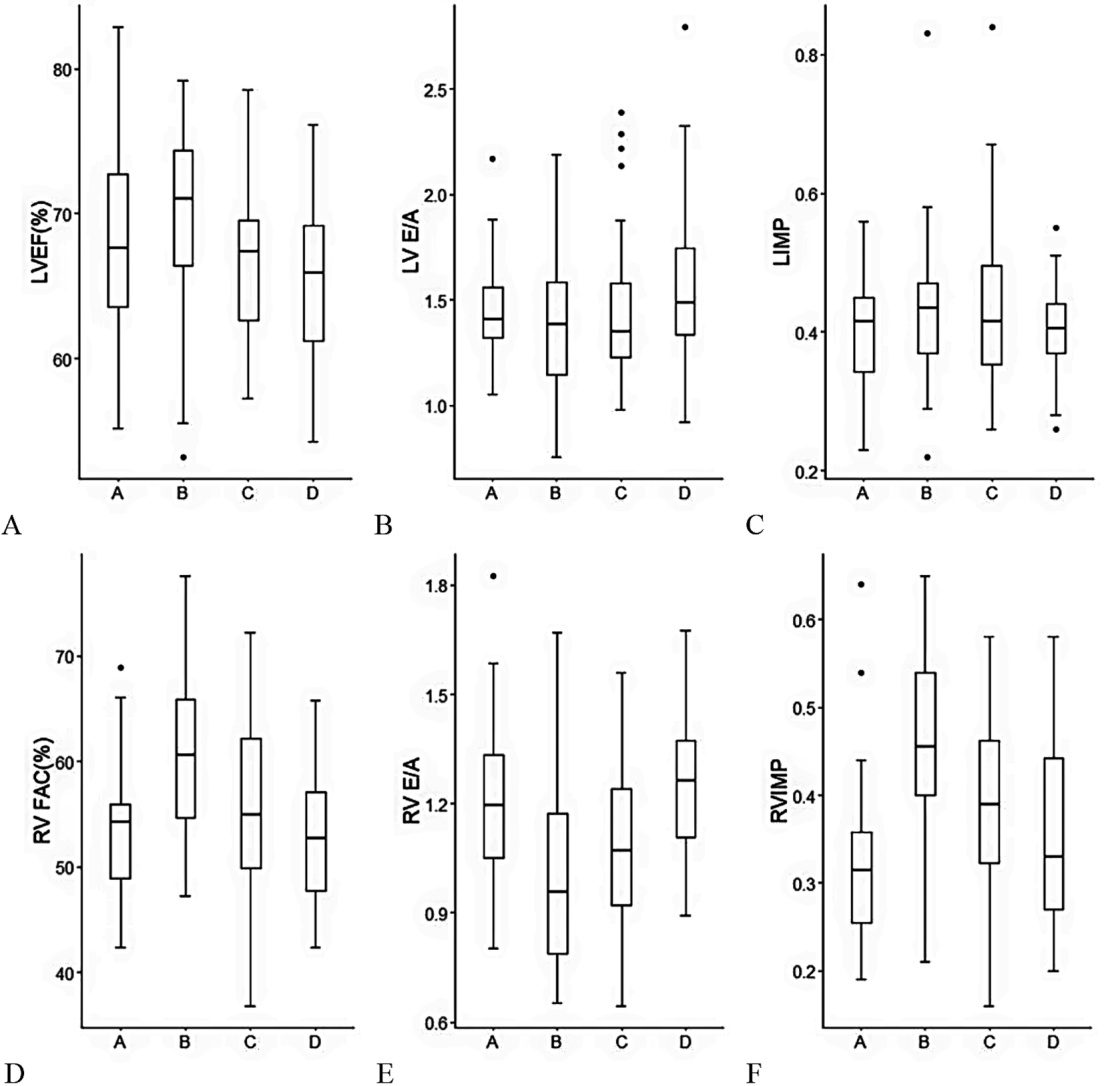
Box plot of LVEF (**A**), LV E/A (**B**), LIMP (**C**), RVFAC (**D**), RV E/A(**E**), RIMP (**F**) during and after labor. LVEF: left ventricle ejection fraction, LV E/A: left ventricular E/A ratio, LIMP: left ventricular index of myocardial performance, RVFAC: right ventricle fractional area change, RV E/A: right ventricular E/A ratio, RIMP: right ventricular index of myocardial performance. In the abscissa, A: the early labor, B: the end of the second stage of labor, C: 2 h postpartum, D: 24 h postpartum respectively

LVEF decreased at 24 h postpartum compared to the end of the second stage of labor (*T* = 3.517, *P* = 0.004) (Fig. 4A). LV E/A and LIMP did not show significant changes (*P* = 0.116, *P* = 0.279) (Fig. 4B and C). From the early labor to 24 h postpartum, RVFAC first increased and then decreased, reaching its maximum at the end of the second stage of labor (*P* = 0.041) (Fig. 4D). RV E/A first decreased and then increased, reaching its minimum at the end of the second stage of labor (*P* = 0.002) (Fig. 4E). RIMP first increased and then decreased, reaching its maximum at the end of the second stage of labor (*P* = 0.002) (Fig. 4F). The heart rate first increased and then decreased, reaching its maximum at the end of the second stage of labor (Table 4).

**Table 4:** Cardiac function during and after labor

Variables	Early labor	End of the second stage of labor	2 h postpartum	24 h postpartum	*P* value
LVEF, %	67.60(9.14)	70.99(7.96)	67.42(6.89)	65.90(7.93)^b^	0.041
LV E/A	1.41(0.24)	1.38(0.44)	1.35(0.35)	1.49(0.41)	0.116
LIMP	0.42(0.11)	0.44(0.10)	0.42(0.14)	0.41(0.07)	0.279
RVFAC, %	54.25(7.00)	60.56(11.19)^a^	54.95(12.29)^b^	52.69(9.28)^b^	0.041
RV E/A	1.20(0.28)	0.96(0.39)^a^	1.07(0.32)	1.26(0.27)^b^	0.002
RIMP	0.32(0.10)	0.46(0.14)^a^	0.39(0.14)	0.33(0.17)^b^	0.002
HR	83.50(17.00)	92.00(14.50)^a^	86.00(12.00)^a^	76.00(83.50)^bc^	< 0.001
Systolic pressure	115.00(8.00)	121.00(5.75)^a^	120.00(7.50)	118.00(5.00)^b^	0.008
Diastolic pressure	72.00(6.75)	74.50(5.50)	72.00(6.00)	71.50(6.50)	0.314

Values reported as median (interquartile range) as appropriate

LVEF: left ventricle ejection fraction, LV E/A: left ventricular E/A ratio, LIMP: left ventricular index of myocardial performance, RVFAC: right ventricle fractional area change, RV E/A: right ventricular E/A ratio, RIMP: right ventricular index of myocardial performance

^a^ Compared with the early labor, the difference is statistically significant, and after q test with Holm correction, *P* < 0.05

^b^ Compared with the end of the second stage of labor, the difference is statistically significant, and after q test with Holm correction, *P* < 0.05

^c^ Compared with 2 h postpartum, the difference is statistically significant, and after q test with Holm correction, *P* < 0.05

ECS had a weak correlation with IVC-CI (*r*=-0.373, *P* < 0.001), LIMP (*r* = 0.298, *P* = 0.022), and RIMP (*r* = 0.211, *P* = 0.021). No correlations were found between ECS and other measurements.

## Discussion

This study used the 28-rib interspaces technique to perform lung ultrasound on healthy singleton-term pregnant women with spontaneous vaginal delivery in a cephalic position. It was observed that some women had a mild EVLW increase at the early labor, the end of the second stage of labor, 2 h postpartum, and 24 h postpartum, which was consistent with the hypothesis. The ECS first increased and then decreased, reaching its peak at 2 h postpartum. This indicated that EVLW accumulated from the early labor to 2 h postpartum, and the risk of pulmonary edema gradually increased. However, from 2 to 24 h postpartum, the EVLW and the risk of pulmonary edema in healthy women were reduced after the compensation of the cardiovascular system.

In a case-control study to assess fluid tolerance and fluid responsiveness in pregnant women with severe pre-eclampsia, Ambrozic et al. [6] examined 12 healthy women in the control group using the 28-rib interspaces technique. No differences were found in ECS between 1 day before delivery, 1 day after delivery, and 4 days after delivery [6]. The main reasons for the difference between their research results and our study may be that: (1) in their control group, 11 of the 12 cases were cesarean sections, while all the cases in our study were spontaneous vaginal delivery; (2) the measurements were obtained 1 day before delivery and 1 and 4 days after delivery in their study, while for our study, measurements were taken at the early labor, the end of the second stage of labor, 2 and 24 h after delivery. According to the results of our study, the period from the end of the second stage of labor to 2 h postpartum, due to the increase in EVLW and the increase in the incidence of pulmonary edema, is the most important monitoring period for women after delivery.

Recently, the evaluation of IVC was used for obstetric monitoring and management [13, 23]. IVC diameter ≤ 2.1 cm and IVC-CI > 50% are considered normal for adults [9]. Our study found that, from early labor to 24 h postpartum, IVC-CI was between 13.36% and 30.77%. It may be due to the persistently high intravascular volume state or the compression of the IVC by the gravid uterus that limited its variation.

In a case-control study of 36 pregnant women with postpartum hemorrhage and 72 healthy controls, the IVC-CI of the controls measured within 2 h after delivery was 18.15% ± 5.07% [13]. In our study, the IVC-CI at the end of the second stage of labor and 2 h postpartum ranged from 13.36 to 30.77%, similar to the results of Massalha et al. [13]. The measurements of Massalha et al. [13] included any time point within 2 h postpartum, while our study only measured at the end of the second stage of labor and 2 h postpartum. Massalha et al. [13] found that using IVC-CI = 27.8% as the cut-off value to predict whether postpartum hemorrhage patients needed a blood transfusion, the sensitivity and specificity reached 75%. Our study suggests that there is a dynamic change in IVC-CI from fetal delivery to 2 h postpartum. When using IVC-CI as a cut-off value, attention should be paid to the measurement time. If the measurement was performed nearly 2 h postpartum, it might lead to a false positive diagnosis due to IVC-CI rebound because of maternal self-compensation. Future research on postpartum IVC should pay attention to the time points of measurement.

Normal adult LVEF is about 63% ± 4%, and RVFAC is about 49% ± 7% [31]. During the spontaneous vaginal delivery of normal healthy women, the LVEF and RVFAC were always higher than those of normal people, suggesting that the ventricular systolic function remained at a relatively high level from the early labor to 24 h after delivery. RVFAC increased at the end of the second stage of labor compared with that at the early labor, indicating that the systolic function of the right ventricle was enhanced. The enhancement of systolic function may be due to the increase in preload caused by the increase in intravascular volume. Under the Frank-Starling mechanism, myocardial contractility was enhanced [23]. The LV E/A of normal adults is about 1.66 ~ 2.13, and the RV E/A is about 1.4 ± 0.37 [31, 24]. During the spontaneous vaginal delivery of healthy women, the LV E/A and RV E/A were always lower than those of normal people, suggesting that the ventricular diastolic function may decline from the early labor to 24 h postpartum (but still within the normal range). At the end of the second stage of labor, the diastolic function of the right ventricle decreased compared to that at the early labor, possibly due to a large amount of venous return.

LIMP < 0.47 and RIMP < 0.43 are considered normal for adults [31, 25]. During the spontaneous vaginal delivery of healthy women, the LIMP did not show significant changes and remained below 0.47, indicating that the left ventricular function remained within the normal range. At the end of the second stage of labor, RIMP increased compared to the early labor, exceeding the critical value of 0.43, indicating abnormal right heart function. The RIMP is calculated using the sum of the isovolumic contraction time and the isovolumic relaxation time and then divided by the ejection time [31]. The causes of abnormal RIMP may be: (1) the prolongation of isovolumic relaxation time due to the decline of right ventricle diastolic function; (2) severe pain and repeated Valsalva maneuver during labor causing sympathetic nervous system excitation and increased afterload, then leading to prolonged isovolumic contraction time and shortened ejection time; and (3) the increase of right ventricular volume causing tricuspid valve regurgitation, then resulting in shortened ejection time. To date, there has been no study using the myocardial performance index to explore changes in maternal cardiac function during childbirth. The results of this study indicate that LIMP and RIMP can be used to evaluate the cardiac function of healthy pregnant women during and after childbirth, and healthy pregnant women may experience cardiac dysfunction during labor.

This study found a positive correlation between IVCi and ECS, a negative correlation between IVC-CI and ECS, and a positive correlation between LIMP, RIMP and ECS. These results suggest that an increase in intravascular volume and a decrease in cardiac function can lead to an increase in ECS, consistent with the hypothesis. The increase in intravascular volume and the rise in right atrial pressure will cause a decrease in venous return from IVC during inspiration, which will lead to an increase in blood retained in the IVC [31, 23]. Therefore, IVCi increases and IVC-CI decreases. Ambrozic et al. [6] found that the increase in EVLW in patients with severe preeclampsia was not only due to increased capillary permeability but also possibly due to volume overload and disturbed left ventricular diastolic function. Unlike patients with severe preeclampsia, the capillary permeability of healthy delivery women is not impaired. Hence, based on the results of our study, the increase in EVLW may be due to increased intravascular volume and decreased left ventricular diastolic function.

Zieleskiewicz et al. [7] found a positive correlation between ECS and E/e’ ratio, which reflected left ventricular diastolic function, indicating a correlation between ECS and parameters measuring cardiac function, consistent with the results of our study. The measurement of E/e’ ratio requires tissue Doppler, but some delivery rooms are not equipped with a transducer that has a tissue Doppler function. Therefore, our study used spectral Doppler to measure LIMP and RIMP.

Recent large observational studies have demonstrated that COVID-19 infection may exhibit abnormal systolic function in echocardiography examinations [27]. And right ventricular involvement may have an impact on pulmonary circulation and, more specifically, the hydrostatic pressures that will affect the balance of intra- and extravascular lung water [4, 28]. In addition, COVID-19 infection can cause lung damage, which will affect lung ultrasound appearances [29, 29]. Therefore, parturients with previous or current COVID-19 infections were excluded from this study.

This study has some limitations. Firstly, the sample size was relatively small due to strict inclusion criteria and high maternal rejection rates. Secondly, due to the included samples being mostly during the daytime and evening before midnight, maternal activity and the use of free positions during this period may be more frequent than after midnight. Hence, the EVLW may be underestimated, leading to enrollment bias. In addition, although the samples included in this study were uncomplicated pregnant women, there was no limitation in the use of epidural anesthesia or oxytocin, which may affect the EVLW and hemodynamics of women. Therefore, our results should be generalized with caution in these circumstances. Finally, this study found that IVC-CI was the smallest at the end of the second stage of labor, indicating a sharp increase in intravascular volume. Interestingly, ECS reached its maximum at 2 h postpartum. This suggested that there may be a time difference between the increase in intravascular volume and the increase in EVLW, which may be the time for fluid transfer. Hence, there may be a lag in the increase in EVLW, which was not considered before this study. Future research may consider using time series analysis to further explore the correlation between intravascular volume and EVLW.

## Conclusions

Bedside ultrasound can be used for monitoring during and after childbirth. The period from the end of the second stage of labor to 2 h postpartum is the critical monitoring period for maternal cardiopulmonary function. The increase in maternal EVLW is correlated with increased intravascular volume and abnormal cardiac function.

### Electronic supplementary material

Below is the link to the electronic supplementary material.

Supplementary Material 1: The changes of B lines at four time points for a woman who underwent spontaneous vaginal delivery

## Data Availability

The datasets used and/or analyzed during the current study are available from the corresponding author upon reasonable request.

## Abbreviations

ECS: echo comet score
EVLW: extravascular lung water
IVC: inferior vena cava
IVC-CI: inferior vena cava collapsibility index
IVCe: end expiratory inferior vena cava diameter
IVCi: end inspiratory inferior vena cava diameter
LIMP: left ventricular index of myocardial performance
LVEF: left ventricle ejection fraction
LV E/A: left ventricular E/A ratio
RIMP: right ventricular index of myocardial performance
RV E/A: right ventricular E/A ratio
RVFAC: right ventricle fractional area change
