# Supplementary material for: Evaluation of extravascular lung water and cardiac function in normal vaginal delivery by intrapartum bedside ultrasound
Source: BMC Pregnancy Childbirth. 2024 Jan 2;24:13. doi: 10.1186/s12884-023-06201-4 (PMC10759567; doi:10.1186/s12884-023-06201-4)
Supplement: Supplementary file 1 — Supplementary Material 1: The changes of B lines at four time points for a woman who underwent spontaneous vaginal delivery [file 12884_2023_6201_MOESM1_ESM.docx]

**Figure S1** The changes of B lines at four time points for a woman who underwent spontaneous vaginal delivery

2 B lines at the early labor


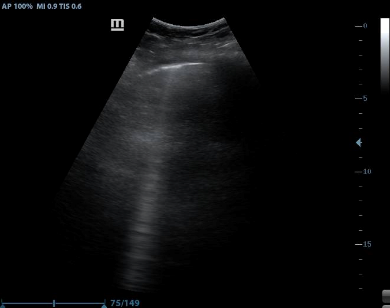

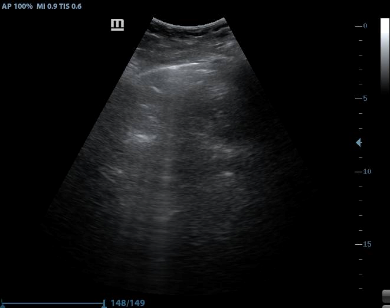


4 B lines at the second stage of labor


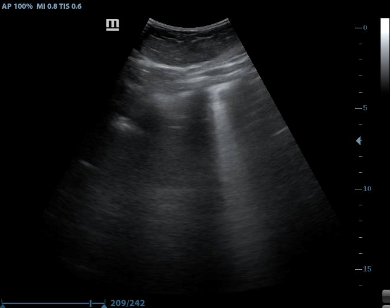

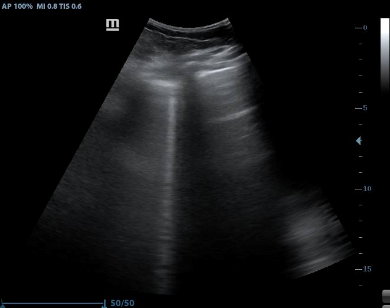

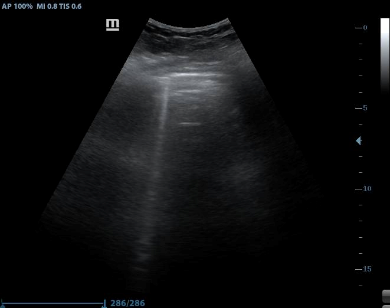

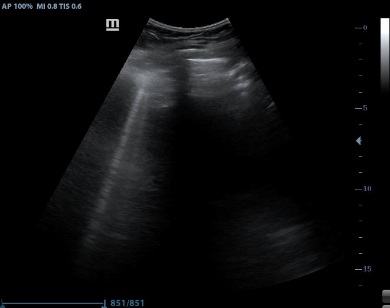


7 B lines at 2 hours postpartum


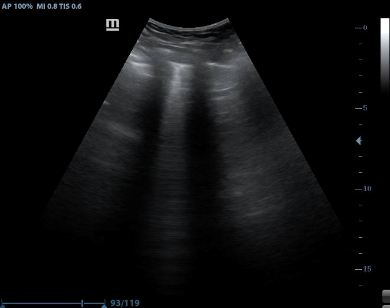

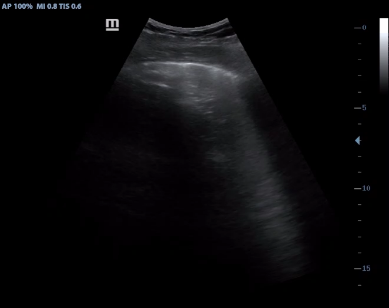

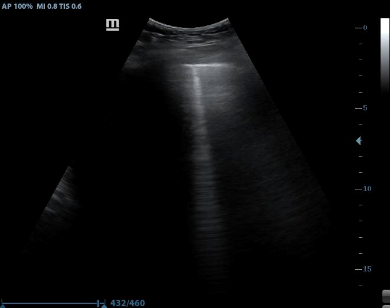

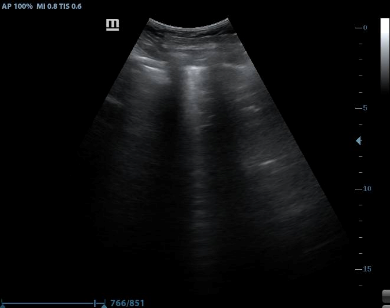

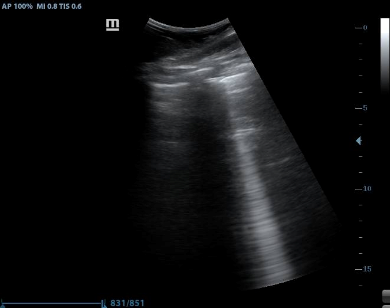

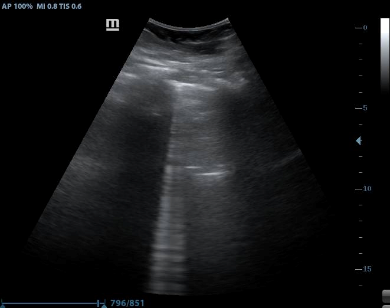
(2 B lines in one view)

2 B lines 24 hours postpartum


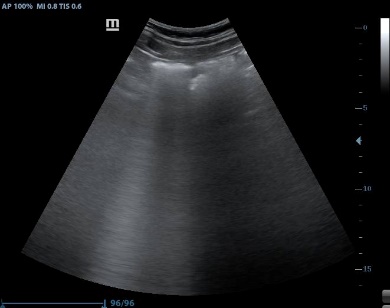
(2 B lines in one view)
